# Changes in Physical Fitness during Summer Months and the School Year in Austrian Elementary School Children—A 4-Year Longitudinal Study

**DOI:** 10.3390/ijerph18136920

**Published:** 2021-06-28

**Authors:** Clemens Drenowatz, Gerson Ferrari, Klaus Greier

**Affiliations:** 1 Division of Sport, Physical Activity and Health, University of Education Upper Austria, 4020 Linz, Austria; 2 Escuela de Ciencias de la Actividad Física, el Deporte y la Salud, Universidad de Santiago de Chile (USACH), Santiago 7500618, Chile; gersonferrari08@yahoo.com.br; 3 Division of Physical Education, Private Educational College (KPH-ES), 6422 Stams, Austria; Nikolaus.greier@kph-es.at; 4 Department of Sports Science, Leopold-Franzens University Innsbruck, 6020 Innsbruck, Austria

**Keywords:** season, academic year, cardio-respiratory endurance, muscular strength, speed, agility, balance, flexibility, body weight

## Abstract

Even though physical fitness is an important component in children’s health and well-being, there has been a decline in physical fitness over the last several decades. The present study examined potential differences in the development of physical fitness during the academic year and summer vacation in Austrian elementary-school children. A total of 214 children (52.3% boys) completed the German motor test in the fall (after summer vacation) and spring (prior to summer vacation) of every grade throughout their elementary school years. This resulted in eight assessments of cardio-respiratory endurance, muscular fitness, flexibility, speed, agility, and balance over a 4-year period. As expected, physical fitness increased with age but the improvement in cardio-respiratory endurance and muscular fitness was more pronounced during summer vacation (*p* < 0.01), while the improvement in speed was more pronounced during the academic year (*p* < 0.01). These results indicate an influence of weather conditions on health-enhancing physical activity in addition to structural and social support. Particularly in geographical regions with cold winters and shorter days, health-enhancing physical activity may be limited. Accordingly, schools need to provide additional opportunities to ensure an adequate amount of physical activity that stimulates the development of physical fitness.

## 1. Introduction

Physical fitness is a key component in human development, and there is considerable evidence on the beneficial association with physical, mental, emotional, and social health, as well as general wellbeing and overall quality of life [1,2,3,4]. Despite these benefits, physical fitness levels in children and adolescents have declined over the last several decades or stabilized at low levels [5,6,7,8]. As physical fitness is defined as the ability to engage in physical activity (PA) and/or exercise [9], along with the fact that fitness levels achieved at young ages are generally maintained into adulthood, this can have a significant impact on future public health [10,11]. While physical fitness increases, in general, with age during childhood and adolescence due to growth and maturation [12,13], this development is influenced by various environmental parameters, including opportunities for fitness-enhancing PA in school and during leisure time, as well as climate and weather conditions.

Several studies have examined changes in physical fitness during the school term and summer vacation with equivocal results [14,15,16,17]. In German children, favorable changes in physical fitness have been observed during the summer months [14], while other studies reported a decline in physical fitness during summer vacation [15,16,17]. The loss of physical fitness during the summer months has been attributed to unfavorable behavioral choices when children are out of school, due to a loss of structure and routine [18]. It should, however, be considered that while providing structure, school days are also characterized by high amounts of sedentary behaviors, particularly if physical education classes and recess are limited [19,20,21]. Accordingly, a longitudinal study showed lower PA during school days compared to weekends, despite a greater freedom for behavioral choices during weekends [22]. There is also evidence for higher PA levels during summer vacation compared to the school term [23]. Other studies, however, reported contrasting results with lower PA during weekend days compared to weekdays [24,25,26], as well as during summer vacation as compared to the school term [16,27].

In addition to daily structure or the lack thereof, other environmental constraints such as climate and weather conditions have been shown to affect behavioral choices [28,29,30,31]. Accordingly, weather conditions may affect intervention strategies targeting PA and physical fitness [28]. Particularly in climates with distinct seasonal differences, warmer weather with low precipitation and longer daylight has been associated with higher levels of moderate-to-vigorous PA [28,29,30], which could contribute to higher physical fitness [14]. There is, however, also the possibility of compromised PA during the summer months when temperatures get too hot, which may occur in subtropical climates [31]. Accordingly, seasonal variation in physical activity and physical fitness depends on several factors that include geographic region, as well as socio-environmental aspects (e.g., access to open space and sports facilities, parental and peer support) [32]. Given the importance of physical fitness for future health and the inconsistent results regarding seasonal changes in physical fitness, additional research on the development of physical fitness in children is warranted. The present study, therefore, examined the changes in various components of physical fitness in Austrian elementary school children during the school year and the summer months over a four-year period.

## 2. Materials and Methods

*Participants.* A convenience sample of 14 elementary schools in the federal state of Tyrol, Austria, were recruited for participation in the study. Due to the longitudinal nature of the study, only first-grade students were eligible to enter the study. Further, participants needed to be able to complete all physical measurements at the time of data collection. Children with physical impairments, therefore, were excluded. Parents provided written informed consent and children provided oral assent at the time of measurement. All study procedures were performed according to the ethical standards of the 2008 Declaration of Helsinki. The study protocol was approved by the Institutional Review Board of the University of Innsbruck, the school authorities of the federal state of Tyrol, and the participating schools.

*Procedure.* Baseline data collection occurred in October 2014 during the school entry evaluation. The first follow-up measurements were taken at the end of grade 1 in June 2014. Similarly, measurements were taken in the fall (after summer vacation) and early summer (before summer vacation) of each grade, with the final measurement occurring in June 2018 (end of grade 4). Accordingly, children were examined a total of 8 times (twice per school year) using similar procedures across all measurement times. All measurements were taken by trained staff in the participating school’s gymnasium during a single visit, which lasted roughly 90 min.

*Measurements.* Body weight (kg) and height (cm) were measured according to standard procedures with children wearing gym clothes and barefoot. Specifically, body weight was measured with a calibrated digital scale (SECA^®^ 803, Hamburg, Germany) to the nearest 0.1 kg and height was measured with a portable stadiometer (SECA^®^ 217, Hamburg, Germany) to the nearest 0.1 cm. Body mass index (BMI) was calculated (kg/m^2^) and converted to BMI percentile (BMIPCT) based on German reference values, with the 90^th^ percentile as the cut-point for overweight/obesity [33].

Subsequently, participants completed the German motor test (DMT6-18) [34]. The DMT6-18 consists of eight test items that measure cardio-respiratory endurance, muscular power, muscular endurance, flexibility, speed, agility, and balance, which are the main components of health-related fitness [9]. Specifically, participants performed a 6 min run, a standing long-jump, push-ups, sit-ups, a stand-and-reach test, a 20 m sprint, sideways jumping, and backwards balancing. All tests were completed according to the specifications provided in the test manual with sufficient resting time between assessments. Following a standardized 5 min warm-up, participants performed the 20 m sprint. Each child completed two trials from a standing starting position with the better attempt being used for further analyses. The remaining tests were performed in random order, except for the 6 min run, which was completed at the end of the testing session in order to avoid undue fatigue. For the push-ups and sit-ups, participants completed two practice trials prior to the assessment, which consisted of single 40 s trials in which as many push-ups and sit-ups as possible needed to be completed, respectively. The standing long-jump consisted of a counter-movement jump with participants being required to land on their feet in order to measure the distance from the take-off line to the heels. No practice trials were allowed but participants were given two attempts, with the better attempt being used in the analyses. There were also two attempts for sideways jumping after 5 practice trials. Participants performed as many jumps as possible for 15 s with the better attempt being used in the analyses. Backwards balancing consisted of walking twice backwards across 6, 4.5, and 3 cm balance beams following two practice trials; the total number of steps completed was recorded. For the stand-and-reach test, participants stood on a bench and bent at the hip in order to reach with their hands as far as possible on a mounted scale while keeping the knees extended. The position needed to be held for 2 s in order to measure the distance from the toes; positive values indicate reaching beyond the toes, while negative values indicate not reaching the toes. Participants completed two attempts without a practice trial. The better attempt was used in the analyses. The 6 min run consisted of a single attempt, in which participants needed to cover as much distance as possible during a 6 min time period.

In addition to raw-performance scores, the DMT6-18 also provides age- and sex-normalized values (z-scores) for each test item. Z-scores below 97 and 92 are interpreted as poor and very poor, respectively, while scores above 103 and 108 are considered good and very good, respectively. Z-scores between 97 and 103 indicate average performance for the respective sex and age group [34].

*Data processing.* Changes in BMIPCT and physical fitness were calculated as the difference between two consecutive measurement times and standardized to the change per month, using the duration between the two measurement times. Measurement intervals from fall to early summer of the next year were used to determine the change in physical fitness during the school term, while measurement intervals from early summer to fall in the same calendar year were used to determine the change during the summer months, when children were out of school. The average across all change values was multiplied by 12 in order to calculate the annual change during the observation period. Further, z-scores in the fall and in the summer were averaged to determine the average performance after the summer holidays and at the end of the school year, respectively. Only children who provided data at all 8 measurement times were included in the subsequent analyses.

*Statistical Analysis.* Data were checked for normal distribution via visual inspection using Q-Q plots. Upon confirmation of the normal distribution, descriptive statistics were calculated and are reported as mean with standard deviation for interval scaled data and frequencies for nominal data. Mixed between-subject within-subject 2 (sex) × 8 (measurement times) ANOVAs were used to examine the change in physical fitness throughout the elementary school years. In addition, differences between the average change in physical fitness during the summer months and during the school term were examined via dependent t-tests for the total sample, and separately for boys and girls. Seasonal differences in age- and sex-standardized performance scores were examined via mixed between-subject within-subject 2 (sex) × 2 (fall vs. spring measurement) ANOVAs. Eta squared (η^2^) was used to determine effect size with values between 0.01 and 0.06 indicating small, values between 0.06 and 0.14 indicating moderate, and values above 0.14 indicating large effect sizes [35]. All statistical analyses were performed with SPSS V26.0 software (SPSS Inc., IBM Corp., Armonk, New York, NY, USA) and a significance level of *p* < 0.05.

## 3. Results

A total of 392 children (55.4% boys) participated in baseline measurements but only 214 children (52.3% boys) provided valid and complete data at all eight measurement times throughout the 4-year observation period. There were no differences in age and anthropometric characteristics at baseline between children included in the analyses and those excluded due to missing data at follow-up measurements. The baseline fitness of children included in the analyses, however, was higher compared to those with incomplete data during follow-up (total z-score 104.8 ± 5.6 vs. 101.8 ± 6.5, *p* < 0.01). Of those included in the analyses, 9.3% were considered overweight/obese at baseline (grade 1). Even though there was a significant sex difference in height, the effect was small and there was no sex difference in BMIPCT. Accordingly, the prevalence of overweight/obesity did not differ between boys and girls. Based on age- and sex-standardized scores, the total sample displayed good overall physical fitness, with very good performance at sideways jumping and push-ups. Only 9.3% displayed poor overall physical fitness, while 32.2% and 27.6% of the participants displayed good and very good overall physical fitness, respectively. Absolute performance was better in boys compared to girls in the 20 m sprint (*p* < 0.01), standing long-jump (*p* < 0.01), sideways jumping (*p* = 0.02), sit-ups (*p* < 0.01), and 6 min run (*p* < 0.01), while girls performed better in the stand-and-reach test (*p* = 0.01). Effect sizes, however, were small to moderate, except for the sex difference in the standing long-jump, where a large effect was observed (Table 1). Based on age- and sex-standardized values, boys performed better than girls at standing long-jump (*p* < 0.01), while girls performed better at sideways jumping and the stand-and-reach test (*p* < 0.03). Effect sizes, however, were small and there was no difference in overall physical fitness between boys and girls.

Even though the mean BMIPCT did not change significantly throughout the observation period, the prevalence of overweight/obesity increased to 16.8%. Further, 2 × 8 ANOVAs indicated a significant improvement in the performance in the individual fitness tests throughout the elementary school years (linear p for trend < 0.01, partial η^2^ ≥ 0.41), except for the stand-and-reach test (Table 2). There were no significant time x sex interaction effects, except for the stand-and-reach test (*p* < 0.01), where a significant improvement was observed for girls (linear *p* for trend < 0.01), while no change was observed in boys.

**Figure 1:**
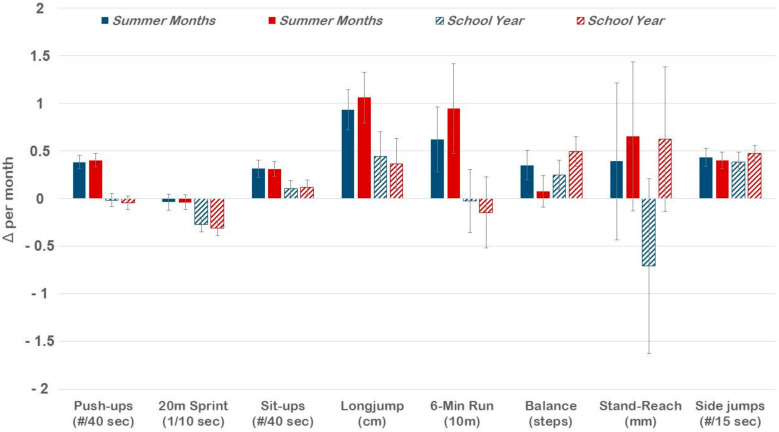
Changes in physical fitness during the school year and the summer months, separately for boys and girls. The values represent the mean change per month with a 95% confidence interval.

The changes in speed, endurance, muscular power, and muscular strength, however, did not occur consistently throughout the year even though no seasonal difference was observed for the change in BMIPCT. Improvements in the 6 min run, push-ups, sit-ups, and standing long-jump were more pronounced during the summer months (*p* < 0.01), while the improvement in the 20 m sprint was more pronounced during the school term (*p* < 0.01). Effect sizes, however, were small, except for the seasonal difference in the 20 m sprint and push-ups, where moderate and large effects were observed, respectively (Table 3). Sex-specific analyses revealed similar results, except for the 6 min run and balancing. The whole-sample seasonal difference in the 6 min run was driven by the greater improvement in girls’ performance during the summer holidays compared to the school term (*p* < 0.01), whereas the seasonal difference for boys was nonsignificant. Similarly, there was a significant seasonal difference in balancing scores for the whole sample due to girls showing a significantly greater improvement during the school year than during the summer months (*p* < 0.01) (Figure 1).

**Figure 2:**
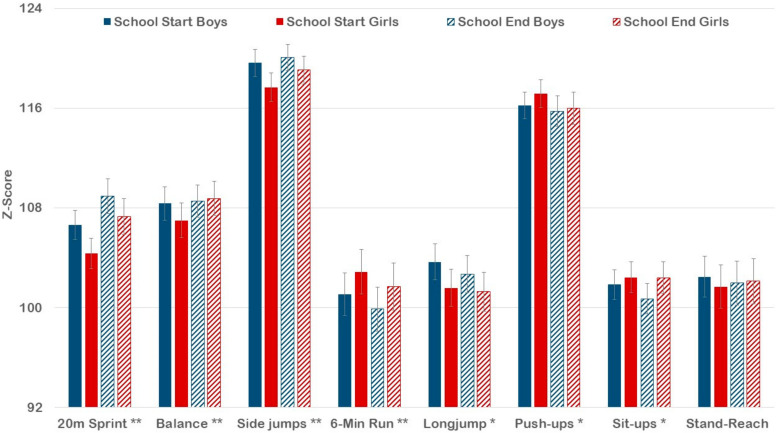
Physical fitness at the beginning and end of the school year based on age- and sex-standardized values for boys and girls. The values represent the mean with a 95% confidence interval. *p* value for difference between school start and end of school year across total sample: * *p* < 0.05, ** *p* < 0.01.

Examining seasonal differences in performance based on age- and sex-standardized scores, there were significant main effects for time across all test items (*p* < 0.05), except for the stand-and-reach test (*p* < 0.05). Performances in the 6 min run, standing long-jump, push-ups, and sit-ups were better in the fall (following summer vacation), while the performances in the 20 m sprint, balancing, and side-ways jumping were better in the summer (at the end of the school year) (Figure 2). Effect sizes of these differences, however, were small, except for a strong effect for sprint. Time by sex interaction effects were also small to non-existent and significant only for balancing and sit-ups (*p* < 0.05). Only girls displayed a better balance performance in the summer as compared to the fall (*p* < 0.01), while sit-ups performance was significantly better during the fall in boys only (*p* < 0.01).

## 4. Discussion

The present study revealed a more pronounced increase in muscular strength and power, as well as cardio-respiratory endurance, particularly in girls, during the summer months compared to the school term. The improvement in speed, on the other hand, was more pronounced during the school term. Despite a higher cardio-respiratory endurance, speed, muscular power, and upper body strength in boys, there were no gender differences in the rate of the development of physical fitness, except for flexibility. No seasonal differences were observed for BMIPCT but overweight/obesity rates increased throughout the elementary school years. Even though effect sizes were small to moderate, these findings can have important implications for future interventions targeting fitness-enhancing PA.

The results are consistent with a study in German elementary school children, which also showed greater improvements in physical fitness during the summer months as compared to the school term [14]. Given the positive correlation between physical fitness and PA [36,37], these results were attributed to seasonal fluctuations in PA. Longer daylight and higher temperatures during the summer have been associated with more PA, while lower temperatures and high precipitation (e.g., rain and snow) during the winter reduce the likelihood for PA participation [30,38]. Specifically, temperatures between 20 and 25 °C have been considered optimal for moderate-to-vigorous PA [39], while temperatures above 30 °C have been associated with lower PA [27,40]. Accordingly, children in temperate climates have been shown to be more active during the summer months [23,41,42,43,44]. In subtropical climates, on the other hand, PA levels have been lower in the summer [27,31], as children tend to stay indoors, which potentially increases sedentary behaviors.

The seasonal difference, however, did not occur consistently across all components of physical fitness. Particularly, energy-based components of physical fitness (e.g., cardiorespiratory endurance and strength), which are important for health [4], improved during the summer months, while the improvement in speed was more pronounced during the school year. Accordingly, the built and social environment needs to be considered in addition to weather conditions. Time and space for free play and access to organized sports along with social support are critical in the facilitation of PA [23,27]. Even though schools are considered an important setting for health-enhancing PA due to the fact that many children can be reached independent of the socio-economic background, the results of the present study indicate that schools may hinder the development of health-related physical fitness. This may be attributed to the fact that the majority of the school day is occupied by sedentary behaviors [16]. In the Austrian elementary school curriculum, physical education is limited to 2 to 3 classes per week (50 min each) and opportunities for active recess are often limited due to lack of space in and around the school. Accordingly, there may not be enough time and opportunities to engage in activities that stimulate metabolic changes, which positively affect cardio-respiratory fitness and muscular strength. PE classes, in most cases, are also conducted by the classroom teacher rather than a PE specialist, which potentially affects the quality of movement experiences provided in PE. The most common activities in PE involve game-like activities that include short bouts, which may have contributed to the improvement in speed during the school year.

With more time and freedom to move, children in the present study appeared to be engaging in more activities that stimulate the development of health-related physical fitness. These results are in contrast to previous research that suggests summer vacation and a lack of daily structure pose significant risks for unfavorable behavioral changes and loss of physical fitness [18,27]. In particular, studies conducted in populations with lower socio-economic background showed a pronounced increase in body fatness and declines in physical fitness during the summer months [16,17], which may be attributed to limited secure space for free play and lack of social support. The predominantly rural environment in the federal state of Tyrol, however, provides a variety of opportunities for safe engagement in health-enhancing PA and indicates that children are selecting active leisure behaviors when it is afforded by the environment. Schools, therefore, need to provide sufficient time and resources for structured and unstructured PA to ensure optimal development of physical fitness in children.

Some limitations of this study, however, need to be considered when interpreting the results. There were no data on PA participation during school days or non-school days, nor data on socio-economic background and living situation, which are critical correlates of physical fitness and leisure time PA. A large number of participants also had to be excluded due to the stringent inclusion criteria of eight consecutive fitness assessments. Further, there was no information on specific weather conditions. Given its geographic location, Austria, however, experiences four distinct seasons, and physical fitness is affected by PA patterns (rather than acute bouts), which are less influenced by acute weather conditions. These aspects also need to be considered regarding the generalizability, and the results may only be applicable to geographical areas with seasonal differences that consist of cold winters and warm summers. On the other hand, the longitudinal, within-subject study design with multiple measurements that span over a four-year period is a considerable strength of this study.

## 5. Conclusions

Regular PA that enhances physical fitness is critical for the development and general health of children and adolescents. As Austrian elementary-school children displayed a greater improvement in health-related fitness in the absence of a daily structure during the summer months, there appears to be a need for the implementation of additional PA opportunities during the school day. Providing a sufficient amount of time and movement experiences to enhance physical fitness in schools may be particularly important during the winter months when recreational activities are limited due to time constraints (e.g., shorter days) and weather conditions.

## Figures and Tables

**Table 1:** Baseline characteristics.

	TOTAL SAMPLE (N = 214)	GIRLS (N = 102)	BOYS (N = 112)	η^2^
Age (years)	6.9 ± 0.4	6.9 ± 0.5	6.9 ± 0.4	0.005
Height (cm) **	122.7 ± 5.5	121.5 ± 5.1	123.7 ± 5.7	0.042
Weight (kg)	24.3 ± 4.0	23.8 ± 3.8	24.8 ± 4.1	0.015
BMI percentile	52.7 ± 27.0	52.5 ± 26.4	52.9 ± 27.6	<0.001
20 m Sprint (s) **	4.76 ± 0.46	4.91 ± 0.48	4.62 ± 0.39	0.103
z-score	102.0 ± 8.9	101.2 ± 9.6	102.7 ± 8.1	0.007
Long-jump (cm) **	114.6 ± 17.1	107.7 ± 14.9	120.9 ± 16.6	0.158
z-score **	102.3 ± 9.3	100.3 ± 9.0	104.1 ± 9.2	0.042
Side jumps (#/15 s) *	23.1 ± 5.5	22.1 ± 4.9	23.9 ± 5.8	0.027
z-score *	114.8 + 10.6	116.6 ± 10.0	112.9 ± 10.9	0.031
Push-ups (#/40 s)	11.7 ± 3.5	11.7 ± 3.6	11.6 ± 3.5	<0.001
z-score	108.1 ± 10.2	108.1 ± 10.2	108.1 ± 10.3	<0.001
Sit-ups (#/40 s) **	15.7 ± 5.1	14.7 ± 5.2	16.5 ± 5.0	0.033
z-score	99.5 ± 7.7	99.4 ± 7.9	99.6 ± 7.5	<0.001
6 min run (m) **	863.4 ± 142.7	827.0 ± 125.6	896.6 ± 149.7	0.060
z-score	102.0 ± 11.9	102.0 ± 12.1	102.1 ± 11.8	<0.001
Stand-reach (cm) *	0.67 ± 5.64	1.69 ± 5.28	−0.27 ± 5.82	0.030
z-score *	101.3 ± 9.5	102.7 ± 9.9	99.8 ± 8.7	0.023
Balance (steps)	27.4 ± 9.9	28.7 ± 9.9	26.3 ± 9.7	0.015
z-score	105.8 ± 10.5	106.0 ± 10.5	105.6 ± 10.7	<0.001
Overall z-score	104.8 ± 5.6	105.5 ± 5.5	104.1 ± 5.7	0.015

Values represent the mean ± SD. *p* value for sex difference: * *p* < 0.05, ** *p* < 0.01, BMI: body mass index.

**Table 2:** Development of BMI percentiles and physical fitness over the 4-year observation period, based on 2 (sex) × 8 (time) ANOVA.

		Wilks λ	p *	Partial η^2^	Annual Change	
					Boys	Girls
Δ BMI percentile	Time	0.943	0.328	0.057	0.53 ± 4.91	0.38 ± 5.15
	Time × Sex	0.970	0.501	0.030		
20 m Sprint (Δ sec/year)	Time	0.216	<0.001	0.784	−0.24 ± 0.15	−0.21 ± 0.13
	Time × Sex	0.962	0.334	0.038		
Long-jump (Δ cm/year)	Time	0.206	<0.001	0.794	7.95 ± 5.33	7.83 ± 7.47
	Time × Sex	00.983	0.827	0.017		
Side jumps (Δ # 15 sec/year)	Time	0.082	<0.001	0.918	5.31 ± 2.05	4.83 ± 3.05
	Time × Sex	0.979	0.737	0.021		
Push-ups (Δ # 40 sec/year)	Time	0.180	<0.001	0.820	1.75 ± 1.60	1.85 ± 1.83
	Time × Sex	0.985	0.861	0.015		
Sit-ups (Δ # 40 sec/year)	Time	0.235	<0.001	0.765	2.40 ± 1.79	2.33 ± 1.85
	Time × Sex	0.948	0.138	0.052		
6 min run (Δ m/year)	Time	0.590	<0.001	0.410	38.33 ± 60.06	29.57 ± 81.27
	Time × Sex	0.936	0.054	0.064		
Stand-reach (Δ cm/year)	Time	0.895	0.122	0.105	0.76 ± 1.77	−0.28 ± 2.03
	Time × Sex	0.874	<0.001	0.126		
Balance (Δ steps/year)	Time	0.228	<0.001	0.772	3.77 ± 2.74	3.54 ± 3.07
	Time × Sex	0.960	0.291	0.040		

Annual change values represent the mean ± SD. * *p* value indicates linear trend for time.

**Table 3:** Changes in BMI percentile and physical fitness during the school year and the summer months.

	Summer Months	School-Year	*p-*Value	η^2^
Δ BMI percentile	0.13 ± 0.91	−0.03 ± 0.90	0.162	0.009
Δ Push-ups (Δ#/month)	0.39 ± 0.37	−0.03 ± 0.37	<0.001	0.276
Δ 20 m sprint (Δs/month)	0.00 ± 0.04	−0.03 ± 0.04	<0.001	0.091
Δ Sit-ups (Δ#/month)	0.31 ± 0.45	0.11 ± 0.41	<0.001	0.059
Δ Long-jump (Δcm/month)	0.99 ± 1.23	0.40 ± 1.35	<0.001	0.057
Δ 6 min run (Δm/month)	7.74 ± 21.16	−0.67 ± 18.22	0.001	0.048
Δ Balance (Δsteps/month)	0.22 ± 0.85	0.37 ± 0.82	0.176	0.009
Δ Stand-reach (Δm/month)	0.05 ± 0.42	−0.01 ± 0.45	0.300	0.005
Δ Side jumps (Δ#/month)	0.42 ± 0.47	0.43 ± 0.50	0.888	<0.001

Values represent the mean ± SD.

## Data Availability

The data presented in this study are available on request from the corresponding author.

## References

[1] Bermejo-Cantarero A., Álvarez-Bueno C., Martínez-Vizcaino V., Redondo-Tébar A., Pozuelo-Carrascosa D.P., Sánchez-López M. (2021). Relationship between both cardiorespiratory and muscular fitness and health-related quality of life in children and adolescents: A systematic review and meta-analysis of observational studies. Health Qual. Life Outcomes.

[2] Janssen I., LeBlanc A.G. (2010). Systematic review of the health benefits of physical activity and fitness in school-aged children and youth. Int. J. Behav. Nutr. Phys. Act..

[3] Morales P.F., López M.S., Moya-Martínez P., García-Prieto J.C., Andres M.M., García N.L., Martínez-Vizcaíno V. (2013). Health-related quality of life, obesity, and fitness in schoolchildren: The Cuenca study. Qual. Life Res..

[4] Ortega F.B., Ruiz J.R., Castillo M.J., Sjöström M. (2008). Physical fitness in childhood and adolescence: A powerful marker of health. Int. J. Obes..

[5] Brunner F., Kornexl E., Kasnter H., Drenowatz C., Greier K. (2021). Fitness trend analysis in male Austrian middle and high school students from 1975 to 2010. Curr. Issues Sport Sci..

[6] Fraser B., Blizzard L., Tomkinson G.R., Lycett K., Wake M., Burgner D., Ranganathan S., Juonala M., Dwyer T., Venn A.J. (2019). The great leap backward: Changes in the jumping performance of Australian children aged 11−12-years between 1985 and 2015. J. Sports Sci..

[7] Müllerová D., Langmajerová J., Sedláček P., Dvořáková J., Hirschner T., Weber Z., Müller L., Brázdová Z.D. (2015). Dramatic decrease in muscular fitness in the Czech schoolchildren over the Last 20 years. Central Eur. J. Public Health.

[8] Tomkinson G.R., Lang J.J., Tremblay M.S. (2019). Temporal trends in the cardiorespiratory fitness of children and adolescents repre-senting 19 high-income and upper middle-income countries between 1981 and 2014. Br. J. Sports Med..

[9] Ruiz J.R., Castro-Piñero J., Artero E.G., Ortega F.B., Sjöström M., Suni J., Castillo M.J. (2009). Predictive validity of health-related fitness in youth: A systematic review. Br. J. Sports Med..

[10] Blasquez Shigaki G., LBarbosa C.C., Batista M.B., Romanzini C.L.P., Gonçalves E.M., Serassuelo Junior H., Ronque E.R. (2020). Tracking of health-related physical fitness between childhood and adulthood. Am. J. Hum. Biol..

[11] Ortega F.B., Cadenas-Sanchez C., Lee D.-C., Ruiz J.R., Blair S.N., Sui X. (2018). Fitness and Fatness as Health Markers through the Lifespan: An Overview of Current Knowledge. Prog. Prev. Med..

[12] Malina R.M., Bouchard C. (2004). Growth, Maturation, and Physical Activity.

[13] Drenowatz C., Greier K., Ruedl G., Kopp M. (2019). Association between Club Sports Participation and Physical Fitness across 6- to 14-Year-Old Austrian Youth. Int. J. Environ. Res. Public Health.

[14] Augste C., Künzell S. (2014). Seasonal variations in physical fitness among elementary school children. J. Sports Sci..

[15] Brazendale K., Beets M.W., Turner-McGrievy G.M., Kaczynski A.T., Pate R.R., Weaver R.G. (2018). Children’s Obesogenic Behaviors During Summer Versus School: A Within-Person Comparison. J. School Health.

[16] Fu Y., Brusseau T.A., Hannon J.C., Burns R.D. (2017). Effect of a 12-Week Summer Break on School Day Physical Activity and Health-Related Fitness in Low-Income Children from CSPAP Schools. J. Environ. Public Health.

[17] Brusseau T.A., Burns R.D., Fu Y., Weaver R.G. (2019). Impact of Year-Round and Traditional School Schedules on Summer Weight Gain and Fitness Loss. Child. Obes..

[18] Brazendale K., Beets M.W., Weaver R.G., Pate R.R., Turner-McGrievy G.M., Kaczynski A.T., Chandler J.L., Bohnert A., Von Hippel P.T. (2017). Understanding differences between summer vs. school obesogenic behaviors of children: The structured days hypothesis. Int. J. Behav. Nutr. Phys. Act..

[19] Da Costa B.G., da Silva K.S., George A.M., de Assis M.A. (2017). Sedentary behavior during school-time: Sociodemographic, weight status, physical education class, and school performance correlates in Brazilian schoolchildren. J. Sci. Med. Sport.

[20] Van Stralen M.M., Yıldırım M., Wulp A., te Velde S.J., Verloigne M., Doessegger A., Androutsos O., Kovács É., Brug J., Chinapaw M.J.M. (2014). Measured sedentary time and physical activity during the school day of European 10- to 12-year-old children: The ENERGY project. J. Sci. Med. Sport.

[21] Egan C.A., Webster C.A., Beets M.W., Weaver R.G., Russ L., Michael D., Nesbitt D., Orendorff K.L. (2019). Sedentary Time and Behavior during School: A Systematic Review and Meta-Analysis. Am. J. Health Educ..

[22] Zhang P., Lee J.E., Stodden D.F., Gao Z. (2019). Longitudinal Trajectories of Children’s Physical Activity and Sedentary Behaviors on Weekdays and Weekends. J. Phys. Act. Health.

[23] Staiano A.E., Broyles S.T., Katzmarzyk P.T. (2015). School Term vs. School Holiday: Associations with Children’s Physical Activity, Screen-Time, Diet and Sleep. Int. J. Environ. Res. Public Health.

[24] Atkin A.J., Sharp S.J., Harrison F., Brage S., van Sluijs E.M. (2016). Seasonal Variation in Children’s Physical Activity and Sedentary Time. Med. Sci. Sports Exerc..

[25] Comte M., Hobin E., Majumdar S.R., Plotnikoff R.C., Ball G.D., McGavock J., MIPASS and Healthy Hearts Investigators Teams (2013). Patterns of weekday and weekend physical activity in youth in 2 Canadian provinces. Appl. Physiol. Nutr. Metab..

[26] Nyberg G.A., Nordenfelt A.M., Ekelund U., Marcus C. (2009). Physical Activity Patterns Measured by Accelerometry in 6- to 10-yr-Old Children. Med. Sci. Sports Exerc..

[27] Volmut T., Pišot R., Planinšec J., Šimunič B. (2020). Physical Activity Drops During Summer Holidays for 6- to 9-Year-Old Children. Front. Public Health.

[28] Turrisi T.B., Bittel K.M., West A.B., Hojjatinia S., Hojjatinia S., Mama S.K., Lagoa C.M., Conroy D.E. (2021). Seasons, weather, and device-measured movement behaviors: A scoping review from 2006 to 2020. Int. J. Behav. Nutr. Phys. Act..

[29] Aibar A., Bois J., Generelo E., Bengochea E., Paillard T., Zaragoza J. (2015). Effect of weather, school transport, and perceived neigh-borhood characteristics on moderate to vigorous physical activity levels of adlescents from two European cities. Environ. Behav..

[30] Rich C., Griffiths L.J., Dezateux C. (2012). Seasonal variation in accelerometer-determined sedentary behaviour and physical activity in children: A review. Int. J. Behav. Nutr. Phys. Act..

[31] Ridgers N.D., Salmon J., Timperio A. (2015). Too hot to move? Objectively assessed seasonal changes in Australian children’s physi-cal activity. Int. J. Behav. Nutr. Phys. Act..

[32] Drenowatz C. (2021). Association of motor competence and physical activity in children—Does the environment matter?. J. Phys. Educ. Sport.

[33] Kromeyer-Hauschild K., Wabitsch M., Kunze D., Geller F., Geiß H.C., Hesse V., Von Hippel A., Jaeger U., Johnsen D., Korte W. (2001). Perzentile für den Body-mass-Index für das Kindes- und Jugendalter unter Heranziehung verschiedener deutscher Stichproben. Mon. Kinderheilkd..

[34] Bös K., Schlenker L., Büsch D., Lämmle L., Müller H., Oberger J., Seidel I., Tittlbach S. (2009). Deutscher Motorik-Test. 6-18 (DMT6-18) [German Motor Abilities Test 6-18 (DMT6-18)].

[35] Cohen J. (1988). Statistical Power Analysis for the Behavioral Sciences.

[36] Dencker M., Thorsson O., Karlsson M.K., Lindén C., Wollmer P., Andersen L.B. (2008). Daily physical activity related to aerobic fitness and body fat in an urban sample of children. Scand. J. Med. Sci. Sports.

[37] Kristensen P.L., Møller N.C., Korsholm L., Kolle E., Wedderkopp N., Froberg K., Andersen L.B. (2010). The association between aerobic fitness and physical activity in children and adolescents: The European youth heart study. Eur. J. Appl. Physiol..

[38] Chan C.B., Ryan D.A. (2009). Assessing the Effects of Weather Conditions on Physical Activity Participation Using Objective Measures. Int. J. Environ. Res. Public Health.

[39] Lewis L.K., Maher C., Belanger K., Tremblay M., Chaput J.-P., Olds T. (2016). At the Mercy of the Gods: Associations Between Weather, Physical Activity, and Sedentary Time in Children. Pediatr. Exerc. Sci..

[40] Tanaka C., Reilly J.J., Tanaka M., Tanaka S. (2016). Seasonal changes in objectively measured sedentary behavior and physical activity in Japanese primary school children. BMC Public Health.

[41] Carson V., Spence J.C., Cutumisu N., Boule N., Edwards J. (2010). Seasonal variation in physical activity among preschool children in a northern Canadian city. Res. Q. Exerc. Sport.

[42] Kolle E., Steene-Johannessen J., Andersen L.B., Anderssen S.A. (2009). Seasonal variation in objectively assessed physical activity among children and adolescents in Norway: A cross-sectional study. Int. J. Behav. Nutr. Phys. Act..

[43] Kristensen P.L., Korsholm L., Møller N.C., Wedderkopp N., Andersen L.B., Froberg K. (2008). Sources of variation in habitual physical activity of children and adolescents: The European youth heart study. Scand. J. Med. Sci. Sports.

[44] Rowlands A.V., Pilgrim E.L., Eston R.G. (2009). Seasonal changes in children’s physical activity: An examination of group changes, intra-individual variability and consistency in activity pattern across season. Ann. Hum. Biol..

